# The Psychological Space of Professionals’ Trust and Distrust in Socio-Technical Systems

**DOI:** 10.11621/pir.2022.0102

**Published:** 2022-03-15

**Authors:** Anna Yu. Akimova, Aleksander A. Oboznov

**Affiliations:** a Lobachevsky State University of Nizhny Novgorod, Nizhny Novgorod, Russia; b Institute of Psychology of the Russian Academy of Sciences, Moscow, Russia

**Keywords:** Socio-technical system, trust, distrust, subject–subject interactions, subject–object interactions

## Abstract

**Background:**

The spatial aspect of professionals’ trust and distrust in socio-technical systems has not been sufficiently explored. The study of its structure, criteria for spatial distribution, and interrelationships of elements is of both scientific and practical interest.

**Objective:**

To perform a comparative analysis of the trust and distrust experienced by professional operators in a socio-technical system of subject–subject and subject–object interactions.

**Design:**

This work is based on A.B. Kupreychenko’s methodological approach to studying trust and distrust in socio-technical systems, adapted by the authors to the railway transport system in Russia. The subjects were 86 locomotive crew members. The main focus was on their trust/distrust in the operation of the socio-technical (railway transport) system, including their workmates, managers, and themselves, as well as the technical objects they operate (locomotives), manufacturers of railway equipment, and conditions of its operation.

**Results:**

The authors identified two relatively independent groups of indicators of trust/distrust in subject–subject and subject–object interactions. Trust in the elements of subject–subject interactions (involving workmates, managers, and the study participants themselves as specialists) was reliably higher than their trust in the elements of subject–object interactions (technical objects, manufacturers of railway equipment, and conditions of its operation). The correlations between trust and distrust in the elements of the socio-technical system were positive.

**Conclusions:**

Trust and distrust perform the functions of integrating/differentiating elements of a socio-technical system according to their predictability in various operating conditions. The degree of trust/distrust in the system elements and their “location” in the space of trust/distrust are important when professionals make decisions in the course of performing professional actions. The results of the study can be used for designing socio-technical systems to increase the predictability of their operation in unstable conditions.

## Introduction

We address the concept of socio-technical systems (STS)^1^ due to a search for effective ways of organizing the labor of teams of professionals who use complex technical devices and technologies to perform socially significant tasks. When designing a specific STS, its developers should consider not only the relationship among its technical devices and processes, but also the relationship between professionals and these devices and processes (subject–object interactions) as well as the relationship among the professionals themselves (subject–subject interactions). Therefore, it is necessary to take into account social and psychological factors, which include professionals’ trust and distrust in the STS being designed (Batchelor & Perkins, 2008; Kupreychenko, 2012; Pozdeeva, 2018; Said, Bouloiz, & Gallab, 2019; Schebel, 2009; Shlyakhovaya, 2018; Winby & Mohrman, 2018; Xiao & Gorman, 2018; Zhuravlev & Lepsky, 2018; and others).

Russian psychologists have discussed the legitimacy of using the concepts of trust and distrust in relation to subject–object interactions—interactions of human professionals with inanimate objects (technical devices and technologies). Some researchers promote the view that relations of trust and distrust arise on the basis of mutual conscious assessments, psychological attitudes, and expectations of people as partners in interaction; therefore, these relations can be considered only in terms of subject–subject interactions (Skripkina, 2015a). Others (including the authors of this article) admit the possibility of applying the concepts of trust and distrust to subject–object interactions, to inanimate objects as artifacts that are created and exist as a result of human activities and which, according to human design, perform certain social functions (Akimova & Oboznov, 2016; Kupreychenko, 2012). These artifacts include technical objects and technologies with which professionals interact in the STS. Our study suggests that professionals may express their trust and distrust both in subject–subject and subject–object interactions.

The structure and interrelationships of trust and distrust in the elements (subjects and objects) of socio-technical systems have been underexplored; therefore, their study is of scientific and practical interest.

The objective of our work was to comparatively study how professional operators’ trust and distrust are manifested in subject–subject and subject–object interactions in the STS.

### The Concepts of Trust and Distrust in Psychology

A significant amount of psychological research has been devoted to the problem of people’s trust and distrust in other people and groups of people, themselves, technical objects, and systems. The current state of the problem has been highlighted in recent scientific papers and analytical review publications (Aldasheva, Zelenova, & Runets, 2020; Antonenko, 2019; Bachmann & Kroeger, 2016; Bodu, 2020; Brattstrem, Faems, & Mehring, 2018; Brion, Lount, & Doyle, 2015; Budyakova, 2017; Chancey, Bliss, & Yamani, 2016; Chapman, Hornsey, & Gillespie, 2020; Miller, Batchelor, & Perkins, 2008; Skripkina, 2017; Strauch, 2016; Tatarko & Nestik, 2019; Woodall & Ringel, 2019; Zinchenko, Zotova, & Tarasova, 2017; and others).

The authors of this article refer to the understanding of trust and distrust that has developed in Russian psychology as conscious personal attitudes towards other people, objects in the world around one, and the world in general (Antonenko, 2020; Kupreychenko, 2008; Skripkina, 2011; and others). The relationships of trust and distrust, according to this understanding, include a person’s ideas about the participants in interaction, expectations associated with this interaction, emotional assessments of interacting subjects and objects, and readiness or unreadiness for certain actions in relation to these subjects and objects. Trust and distrust perform a number of functions in various spheres of human life, including the integration or disintegration of people’s relationships with themselves, those around them, and the world as a whole; a decrease or increase in the stress level in human interactions; regulation of interpersonal and group interactions; etc.

Theoretical and empirical studies have indicated that trust and distrust arise in situations of uncertainty associated with diversity of choice of further actions and difficulty in predicting the behavior of a subject or object interacting with a person (Kupreychenko, 2008; Skripkina, 2015a; and others).

A number of factors of trust and distrust have been identified. Of particular importance are those that reflect the features and significance of the interaction situation, the characteristics of the object of trust, and the personal characteristics of the subject of trust (Aldasheva, et al., 2020; Antonenko, 2019; Kupreychenko, 2008; Skripkina, 2015a; and others). In relation to interactions with the socio-technical system, such factors include a person’s ideas about the reliability of all components of the system, knowledge about the functioning of technical elements and the competence of those managing the system, experience in working with the system, assessment of one’s own capabilities, individual characteristics (Akimova, 2013; Kupreychenko, 2012; Strauch, 2016; and others).

Trust and distrust in another person or an object can be manifest in two ways: as inversely dependent or independent relationships. In the first case, if trust in a person or an object increases, distrust decreases, and vice versa. With extremely high trust, distrust becomes zero. In other words, these relationships can be mutually exclusive. In the second case, any combination of trust and distrust is possible—e.g., simultaneously expressed both trust and distrust in the same person, an ambivalent attitude. In the space of manifestations of trust and distrust, we can distinguish between the area in which they act as dependent relationships and the area in which they appear as independent relationships (Kupreychenko, 2008).

### Psychological Space of Professional Operators’ Trust and Distrust in the STS

We can explore the spatial aspect of professional operators’ trust and distrust in the STS due to their psychological relationships, which are “an integral system of individual, selective, conscious personal connections with various aspects of objective reality” (Myasishchev, 1957, p. 143). These connections are formed during interactions of professionals with the elements of the STS: the technical objects they operate, as well as their workmates, managers, service personnel, equipment developers, etc.

According to researchers of the spatial aspect of psychological relationships (Pozniakov, 2015; Skripkina, 2015b; Zhuravlev & Kupreychenko, 2012; and others), trust and distrust in various elements of the STS (professionals’ trust and distrust in the technical objects; trust and distrust in the people who ensure the system’s operation, such as workmates, managers, and service personnel; trust and distrust in equipment developers such as manufacturers; trust and distrust in themselves as professionals; etc.) form the space of professionals’ trust and distrust in the STS.

The analysis of this space can be associated with the localization of ideal images of the system elements as objects of trust and distrust in the professionals’ subjective space.

The very idea of “space”, according to the Russian psychologist V.P. Pozniakov, is embedded in the understanding of psychological relationships as manifestations of a person’s “internal” position in interaction with objects and subjects of the surrounding world (Pozniakov, 2015). Interactions of professionals with the elements of their STS are largely determined by their idea about the importance of these elements for the successful operation of the system (they may regard some elements as very significant and disregard or deliberately ignore others). It therefore seems reasonable to consider the subjective significance of an object or subject of trust and distrust, using as a criterion their location in the space of professionals’ trust and distrust in the STS. Thus, the more significant elements of the STS can be presented in their experiences as “closer” ones, deserving of higher trust. Less significant ones appear to be “more remote”, trust in which is lower (or even distrust is expressed towards them).

*On the basis of the above, we formulated the following research hypotheses:*

– The space of professional operators’ trust and distrust in the STS is differentiated and includes trust and distrust in the system in subject–subject and subject–object interactions; and
– Professional operators’ trust and distrust in the elements of the STS in subject–subject interactions differs from their trust and distrust in the elements of the STS in subject–object interactions.

## Methods

### Content of the Validated Material

This work was based on the methodological approach to studying trust and distrust in socio-technical systems proposed by A.B. Kupreychenko (2012). The main focus was on the general properties and elements of trust and distrust, which were considered the interconnected and opposite poles of a single concept. Focusing on the above consideration of trust and distrust, we will refer to them as “trust/distrust”.

According to this approach, the following elements can be distinguished in the structure of trust/distrust in the socio-technical system:

– trust/distrust in the system organization and operation;
– trust/distrust in individual functional modules (hierarchical levels, material and technical base, technologies, individual units and elements);
– trust/distrust in various categories of people who ensure the system’s operation (organizers, moderators of the system, and other interested parties);
– trust/distrust in oneself as a professional or user; and
– trust/distrust in the system’s operating conditions.

We studied the space of trust/distrust of locomotive crew members in the elements of the socio-technical system of railway transport (hereinafter referred to as “the railway system”). The components of the railway system include: trains; infrastructure (centralized control systems, level crossings, railway stations, etc.); locomotive crew members directly managing the trains; dispatchers providing remote control of railway flows; workers and specialists serving the structure of railway transport (train builders, electromechanics, station attendants, engineers, technologists, managers, etc.); developers and manufacturers of railway transport, etc.

Locomotive crews, which directly operate trains, are a key component of the railway system and determine its overall performance. The specificity of their activities allows them to be considered professional operators (Nersesyan, 1992). In previous studies, the authors of this article obtained data on trust and distrust among locomotive crew members using railway equipment under operating conditions, and a correlation was established between the effectiveness of activities and the degree of trust in the technology (Akimova, 2013; Akimova & Oboznov, 2016; and others).

### Participants

The study involved 86 locomotive crew members. The sample was formed by random selection from the general population of crews. The data on the study participants are presented in *Table 1*.

**Table 1:** Data on the study participants

Indicator name	Indicator value	
	Number	%
Total sample size	86	100
Gender		
Male	86	100
Female	0	0
Age groups		
18–30 years	29	33.7
31–40 years	15	17.5
41–50 years	26	30.2
51–60 years	16	18.6
Position		
Operator	58	67.4
Assistant operator	28	32.6
Work experience in the position		
Up to 10 years	46	53.4
11–20 years	19	22.1
21–30 years	12	13.9
More than 30 years	9	10.6

### Procedure

To assess the trust/distrust of locomotive crew members (hereinafter referred to as “operators’) in the railway system, the authors used a questionnaire based on the methodological approach proposed by A.B. Kupreychenko (2012) and adapted to the railway transport system. The questionnaire contained 36 statements to assess six indicators of trust/distrust in the socio-technical system: five indicators — Reliability, Predictability, Attachment, Identity, and Prudence — were assessed by the participants using bipolar trust/distrust scales, and the Hazard indicator, related only to distrust, was assessed according to a monopolar scale. The participants assessed their trust/distrust in the following elements of the railway system: 1) the technical object (locomotive); 2) the operating conditions of the equipment (locomotive), 3) themselves; 4) another locomotive crew member (workmate); 5) the direct supervisor (operator-instructor), and 6) railway equipment manufacturers.

Here are some examples of the questionnaire statements:

“The locomotive I work on is reliable

“The operator-instructor’s actions are predictable.

They were instructed to rate the degree of agreement with the statements on a 5-point scale: from 1 (strongly disagree) to 5 (strongly agree).

Data were collected by the researchers in person. A preliminary conversation was held with the participants about the purpose of the research, as well as about the anonymity of the subsequent use of the results. Then the participants were presented with a form with the stimulus material of the questionnaire, on which they recorded in writing the degree of agreement with the statements. The duration of the study was 20–30 minutes. Statistical data processing was carried out using the SPSS Statistics 22 software.

## Results

Taking into account the validity of using the categories of space in relation to the concepts of trust and distrust, a hierarchical cluster analysis of the research results was carried out (the intergroup communication method, with the measure of “proximity” being the squared Euclidean distance). Subjected to clustering were the indicators of trust/distrust placed by the participants in their workmates, managers, and themselves in the context of the railway transport system’s operation, as well as the technical objects (locomotives), manufacturers of railway equipment, and its operating conditions (see *Figure*).

The cluster analysis revealed two clusters of indicators of trust/distrust in the elements of the socio-technical system.

The first cluster includes the indicators of the operators’ trust/distrust in their workmates, managers, and themselves as specialists. Within this cluster, the “closest” to each other — i.e., “similar” in the subjective assessment of the participants — are the indicators of trust/distrust in their workmates and themselves (cluster distance = 760). Less “close” to these indicators is the indicator of trust/distrust in their managers (cluster distance = 1,021).

**Figure.**
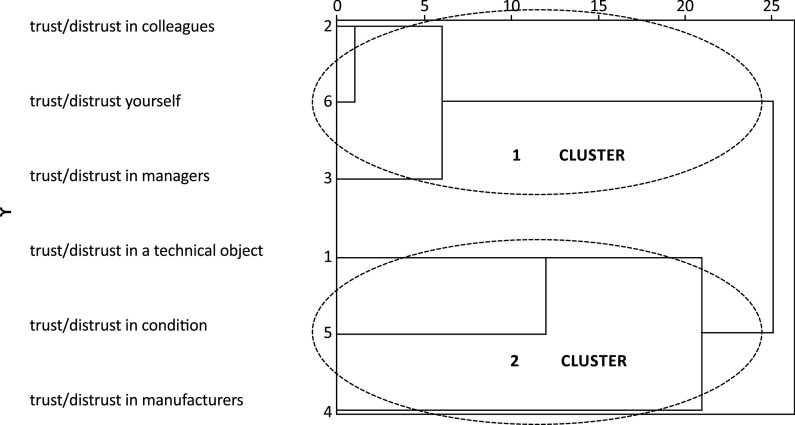
The results of hierarchical cluster analysis of the indicators of the operators’ trust/distrust in the elements of the railway transport system

It should be emphasized that the above indicators are “the closest” to each other, “the most similar” as compared to any indicators included in the second cluster (inter-cluster distance = 1,752).

The second cluster includes indicators of trust/distrust in the technical object, its operating conditions, and equipment manufacturers. Within this cluster, “the closest” to each other are the indicators of trust/distrust in the technical object and its operating conditions (cluster distance = 1,199); somewhat “more remote” from them are the indicators of trust/distrust in the railway equipment manufacturers (cluster distance = 1,309).

Comparing the average values of trust/distrust in the elements of the socio-technical system clearly shows that the locomotive crew members who took part in the study put significantly more trust in their workmates, managers, and themselves (according to the indicators of the first cluster) than in the technical objects (locomotives), manufacturers of railway equipment, and its operating conditions (according to the indicators of the second cluster) (see *Table 2*). The differences between the closest values of trust/distrust in the elements belonging to the different clusters (managers and technical objects) are statistically significant according to the Mann-Whitney U test (U = 2,857.5, significance level p = .01).

**Table 2:** Indicators of trust/distrust in the elements of the railway transport system identified by the locomotive crew members

Element of the railway transport system	Indicators of trust/distrust in of the railway transport the elements system	
	Mean	SD
Workmates	23.45	2.53
Operators themselves as professionals	22.92	2.62
Managers	22.91	3.25
Technical objects (locomotives)	21.40	3.71
Equipment operating conditions	21.38	3.41
Equipment manufacturers	20.81	3.98

The validity of identifying two groups of indicators in the space of trust/distrust in the railway transport system was confirmed by factor analysis (principal components analysis) with varimax rotation. Two relatively independent factors were identified, describing 58.67% of the explained variance. Factor loadings less than .60 were excluded from further meaningful analysis (see *Table 3*).

**Table 3:** Results of the factor analysis of trust/distrust in the railway transport system conducted among the locomotive crew members

Element of the railway transport system	Factor loadings of of trust/distrust in system indicators elements	
	Factor 1	Factor 2
Workmates	.800	
Operators themselves as professionals	.662	
Managers	.722	
Technical objects (locomotives)		.773
Equipment operating conditions		.805
Equipment manufacturers		.669

The first factor included indicators of trust/distrust in the workmates, managers, and study participants themselves. The explained cumulative variance of the first factor was 29% of the original correlation matrix. The second factor includes indicators of trust/distrust in the technical objects, equipment manufacturers, and equipment operating conditions. The explained cumulative variance of the second factor was 30% of the original correlation matrix.

Thus, the indicators of trust/distrust in the elements of the socio-technical system form a structure of two relatively independent factors. Within each factor, the interrelationships of the components are stronger than the interrelationships with the components of the other factor.

A correlation analysis was also carried out (using Spearman’s rank correlation coefficients) to determine all the existing relationships of trust/distrust in the system elements (see *Table 4*).

**Table 4:** Results of the correlation analysis of trust/distrust in the elements of the railway transport system

Element of the railway transport system	Operators themselves as professionals	Managers	Technical objects	Equipment operating conditions	Equipment manufacturers
Workmates	.382**	.393**		.280**	.159*
Operators professionals themselves as		.369**	.273*	.250*	
Managers				.254*	.382**
Technical objects				.466**	.238*
Operating conditions					.406**

Note. * p <.05; ** p < .01; the table shows only statistically significant rank correlation coefficients.

According to the correlation analysis, there were 12 statistically significant correlations out of 15 possible (80%); all the correlations were positive. Thus, in the space of the operators’ trust/distrust in the STS, the change in trust/distrust in most elements of the system occurs in a coordinated manner (either as an agreed increase or as an agreed decrease).

Furthermore, despite being assigned to different groups, trust/distrust in the operators themselves as professionals is directly interconnected with trust/distrust in the technical object and equipment operating conditions, whereas trust/distrust in workmates and managers is interconnected with trust/distrust in the manufacturers of railway equipment and its operating conditions.

## Discussion

Prior studies of trust/distrust in technical systems have focused on the level of a professional’s trust in these systems, largely determined by the characteristics of the systems (Hancock et al., 2011; Hancock, Kessler, Kaplan, Brill, & Szalma, 2021; and others). The level of trust, according to the available data, determined the effectiveness of the actions performed (Kraus, Scholz, Stiegemeier, & Baumann, 2020; Lee & See, 2004; Verberne, Ham, & Midden, 2015; and others). However, modern technology, becoming more autonomous and “intellectual”, performs roles more similar to those of members of a production team. The result of the work of such a team is more determined by interaction (coordination, cooperation, etc.) than by the reliability and serviceability of equipment (Adams, Breazeal, Brooks, & Scassellati, 2000; Sheridan, 2016; Shneiderman, 2020; and others). According to Chiou and Lee (2021), further study of trust/distrust in technical systems should be based on a relational approach. This assumes that all participants in the interaction (specialists, technical system, production situations) are inextricably linked and act as its integral components.

Agreeing with this statement, we examined the structure of relationships of professional operators’ trust/distrust in the elements of a socio-technical system, in which all these elements (including the professional operators themselves) are parts of this system.

The results of the study indicate that the subjective space of the operators’ trust/ distrust in the elements of the STS are located in different ways and form two groups of objects. The objects within each group are “closer” to each other than to the objects in the other group. Considering that the “closeness–remoteness” of their location reflects the subjective significance of the object of trust/distrust for the operators, we can make the following assumptions.

In the space of trust/distrust in the STS, those elements that belong to the same group are similar in subjective significance: first, the relationships of trust/distrust in the operators themselves, their workmates and managers, which can be attributed to subject–subject interactions; and, second, the relationships of trust/distrust in the technical objects (locomotives), manufacturers of railway equipment, and its operating conditions), which can be attributed to subject–object interactions.

The selected groups of trust/distrust in the elements of the STS have different subjective significance for the operators and are, according to the factor analysis, relatively independent. Consequently, the space of trust/distrust in the STS is structured and forms two relatively independent components, meaningfully reflecting the relationships of professional operators’ trust/distrust in subject–subject and subject–object interactions.

It should be emphasized that the operators who took part in the study put significantly more trust in the elements of the first group than in those of the second group.

The data obtained in the study are consistent with those from a study on the structure of conceptual models among nuclear power plant (NPP) operators included in the corresponding STS operation (Oboznov, Chernetskaya, & Bessonova, 2017). In that study, it was shown that the operators consider the operation of plant units as a man–machine complex, which is characterized by numerous intrasystem and intersystem interactions, including nonlinear and unstable ones, non-stationary extreme conditions of the working environment, etc. It was noted that most of the professionals are of the opinion that when one of the characteristics of a technical system changes, the change in other characteristics is difficult to predict. At the same time, the majority of NPP professionals believe that joint involvement in activities increases the predictability that all services will properly perform their duties, thereby ensuring the stable operation of the entire system.

Taking into account the similarity of the basic patterns of functioning of various socio-technical systems, we can assume that the conclusions of the NPP study, in general, are applicable to the railway transport system. This allows us to conclude that the trust/distrust of locomotive crew members in the elements of their STS in its spatial aspect performs the functions of integrating/differentiating the elements according to the degree of predictability of their behavior in various operating conditions. Subjectively, more predictable and therefore more trustworthy, are elements that are “the closest” to each other. Such relationships are those of trust/distrust in oneself, one’s workmates, and managers. “More remote” and subjectively less predictable are the relationships of trust/distrust in the technical object, its operating conditions, and manufacturers.

After conducting a meta-analysis of more than 230 studies of operators’ trust/ distrust in automated systems, conducted from 1974 to 2021, American researchers John D. Lee and Erin Chiou concluded that the essence of performance in systems associated with the management of complex equipment is not to maximize or even “calibrate” trust, but to maintain the trust process for more sustainable partnerships between humans and automated processes (Chiou & Lee, 2021).

Our study provided empirical confirmation of this conclusion. A significant number of positive relationships of operators’ trust/distrust in the elements of the railway system were shown to be determined, indicating the agreed nature of the change—an increase or decrease—in trust/distrust in these elements. On the other hand, it was shown that, despite the differentiation of trust/distrust in the elements of the system according to the degree of their subjective significance, this nature of their interrelationships reflects the integrity of these attitudes towards all the elements of the STS and the subjective importance of each element for the operators in ensuring the system’s operation.

## Conclusion

The study confirmed our hypotheses. First, it was found that trust/distrust in individual elements of the STS forms a structured space of trust/distrust in the entire system. This space is differentiated and includes two groups of relatively independent relationships of trust/distrust in subject–subject and subject–object interactions within the system. Second, the trust that professional operators have in the STS elements in subject–subject interactions that form the first group is significantly higher than their trust in the STS elements in subject–object interactions that form the second group. The “location” of the indicators of trust/distrust in the system elements within the space of trust/distrust that professional operators have in the STS is largely determined by the subjective significance of the elements for the predictability of the STS operation.

It can be assumed that the relationships of trust/distrust perform the functions of integrating/differentiating the elements according to the degree of predictability of their behavior in various system operating conditions and, therefore, play an important role in professionals’ decisions about their professional actions in various interactions with the system elements.

Positive relationships of professional operators’ trust/distrust in the STS elements were determined: trust in most elements of the system changes (increases or decreases) consistently. Such interrelationships probably reflect the integrity of trust/distrust in all the system elements, as well as the importance of each element in ensuring the system’s operation.

The results of the study can be used for designing socio-technical systems, in order to include trust in both subject–subject relations between professionals, and subject–object relations—the attitude of professionals to technical objects. In the latter case, a promising direction is the development of interfaces that are intuitive for professionals, the inclusion of decision support systems (particularly artificial intelligence), as well as the design of ergonomic and comfortable workplaces. In addition, the data on relationships trust/distrust in various elements of the system obtained in the research can find practical application in training of professionals who make decisions on managing complex technical objects in various working conditions. In particular, assessing trust in elements of the socio-technical system can be introduced into the professional training program, and the results of the assessment can be used to adjust training programs. For example, the implementation of continuous development programs for professionals will contribute to the growth of their competence and, consequently, increase their self-confidence. As a result, such professionals increase the degree of trust in subject–subject and subject–object interactions. In the first case, this leads to the optimization of interactions within the work team, the maintenance of a favorable socio-psychological climate, and consequently, a more coordinated and efficient implementation of production tasks. In the second case, it can reduce the subjective complexity of the working situation, mitigate tension in difficult and dangerous situations, and, in general, facilitate appropriate actions in operating technical objects.

## Limitations

The research results should be considered within the theoretical framework not of a separate role of trust/distrust in technology, people (professionals, managers), and other factors, but specifically in terms of trust/distrust in the socio-technical system, which is characterized by the functional relationships of all these elements.

This is the novelty of the study and, at the same time, a limitation on the application of its results to socio-technical systems, but not to social or technical ones taken separately. The research data were obtained on a sample of locomotive crew members in railway transport.

Possible restrictions on the use of the results may be associated with the lack of data on the gender specificity of trust/distrust in socio-technical systems, since all the study participants are men. In addition, the object of the study was the railway transport system. It can be assumed that the structure of the space of trust/distrust in other socio-systems will have its own characteristics. At the same time, the results and further conclusions are applicable, in our opinion, to professional operators of other types of long-term operation vehicles.

## Footnotes

1 The concept of *socio-technical systems* was introduced in the 1960s by E. Trist and F. Emery from the Tavistock Institute of Human Relations (UK).
